# Elevated Serum Soluble CD137 in Inflammatory Bowel Disease and Primary Sclerosing Cholangitis Highlights a Shared Immunoregulatory Signature

**DOI:** 10.3390/biomedicines14071483

**Published:** 2026-06-30

**Authors:** Tanja Elger, Muriel Huss, Johanna Loibl, Patricia Mester, Petra Stoeckert, Arne Kandulski, Martina Müller, Hauke Christian Tews, Christa Buechler

**Affiliations:** Department of Internal Medicine I, Gastroenterology, Hepatology, Endocrinology, Rheumatology, and Infectious Diseases, University Hospital Regensburg, 93053 Regensburg, Germany; tanja.elger@klinik.uni-regensburg.de (T.E.); muriel.huss@klinik.uni-regensburg.de (M.H.); johanna.loibl@klinik.uni-regensburg.de (J.L.); patricia.mester@klinik.uni-regensburg.de (P.M.); petra.stoeckert@klinik.uni-regensburg.de (P.S.); arne.kandulski@klinik.uni-regensburg.de (A.K.); martina.mueller-schilling@klinik.uni-regensburg.de (M.M.); hauke.tews@klinik.uni-regensburg.de (H.C.T.)

**Keywords:** autoimmune disease, fecal calprotectin, fibrosis score, MELD score, IBD, PSC, ulcerative colitis, Crohn’s disease

## Abstract

**Background**: Soluble CD137 (sCD137) has immunoregulatory properties and is increased in chronic inflammatory and autoimmune diseases, as well as in liver cirrhosis. Its relevance in inflammatory bowel disease (IBD) and primary sclerosing cholangitis associated with IBD (PSC-IBD) remains unclear. We measured serum sCD137 levels in IBD, PSC-IBD, PSC without IBD, and metabolic dysfunction-associated steatotic liver disease (MASLD, as a liver disease control) and assessed whether sCD137 is associated with intestinal inflammatory activity or liver disease severity. **Methods**: Serum sCD137 levels were measured in 77 patients with IBD, 33 with PSC-IBD, 11 with PSC without IBD, 26 with MASLD, and 78 healthy controls. In IBD, associations with serum C-reactive protein, fecal calprotectin, and symptom burden were examined. In PSC and PSC-IBD, associations with liver dysfunction and fibrosis stage, assessed by measurement of liver stiffness, were analyzed. **Results**: Serum sCD137 levels were higher in IBD than in controls, with similar levels in Crohn’s disease and ulcerative colitis. In IBD, sCD137 was not associated with C-reactive protein, fecal calprotectin, stool consistency, symptom extent, disease duration, or disease localization. Serum sCD137 levels were higher in PSC/PSC-IBD than in controls, patients with MASLD, and patients with isolated IBD. Patients with PSC without IBD showed similar levels to those with PSC-IBD. In PSC/PSC-IBD, serum sCD137 levels did not increase with a higher fibrosis stage. **Conclusions**: Serum sCD137 is a disease-associated systemic immune marker in IBD and PSC, with a stronger signal in PSC-associated disease than in isolated IBD. However, sCD137 does not reflect intestinal inflammatory activity or liver fibrosis severity in this cohort, suggesting that it captures disease-associated immune dysregulation rather than current inflammatory or fibrotic burden.

## 1. Introduction

Inflammatory bowel diseases (IBDs) are characterized by chronic inflammation of the intestinal mucosal lamina propria [1,2]. Tumor necrosis factor (TNF) is a key cytokine in IBD that has been shown to induce epithelial cell death, thereby exacerbating disease severity [3,4]. Approved for use in IBD almost 25 years ago, anti-TNF therapy achieves remission in about 70% of patients [4].

The CD137 ligand (CD137L) belongs to the TNF superfamily and is expressed on antigen-presenting cells [5]. CD137L has been shown to increase the TNF levels of macrophages by activating toll-like receptors [6]. CD137L usually acts in concert with CD137, a member of the TNF receptor family [7,8], but for this effect, CD137 was not involved [6].

Both CD137 and CD137L are expressed by immune cells and regulate leukocyte proliferation, differentiation, activation, and survival. The ligand is a transmembrane protein that can also transfer signals into the cell [8,9,10]. Activation of CD137 by antibodies causes macrophages to polarize to an M1 type and induces the synthesis of inflammatory cytokines [11].

The activity of CD137 is regulated by a soluble variant, soluble CD137 (sCD137) [9], which is produced by differential splicing of CD137 or shedding via a disintegrin and metalloproteinase 10 [12,13,14]. Soluble CD137 can disrupt CD137 binding to CD137L, likely exerting inhibitory effects on this signaling pathway [9,12]. Recombinant sCD137 mitigates the polarization of both T helper 1 and 2 cells, thereby lowering TNF levels in the cell culture medium [15]. The anti-inflammatory effect of sCD137 has also been reported in macrophages, where it reduces IL-6 and TNF levels [16]. Recombinant sCD137 induces CD4+ T cell anergy and reduces antigen-specific T cell proliferation and inflammatory cytokine production by CD8 effector memory T cells [17].

T cells and macrophages play a key role in the development of IBD [18,19,20]. T helper 1, 2, and 17 cells contribute to intestinal inflammation [20], and the function of intestinal macrophages, a heterogeneous cell population, becomes dysregulated in IBD [21].

CD137 is expressed on activated intestinal T lymphocytes [22], and stimulation with anti-CD137 antibodies enhances T-cell cytotoxicity and proliferation of these cells [22]. CD137 is also expressed on mononuclear cells in the lamina propria of patients with Crohn’s disease and, to a lesser extent, in those with ulcerative colitis. However, it could not be detected in the control tissue [23].

The pathological role of CD137-CD137L signaling in IBD has been addressed in previous studies. One of these studies used a colitis model based on the transfer of naïve CD4+ T cells [24]. However, severe combined immunodeficient (SCID) mice reconstituted with wild-type or CD137^−^/^−^CD45RBhiCD4^+^ T cells exhibited similar levels of disease activity. The wild-type reconstituted mice exhibited a T helper cell 1 response, whereas the mutant transfer model showed a mixed T helper cell 1/2 response. This study showed that CD137 expression on CD4+ T cells is not essential for disease development and progression [24]. In an acute dextran sodium sulfate (DSS)-induced colitis model, both CD137-/- and wild-type mice exhibited comparable disease activity [25]. In this model, the resolution of inflammation was impaired in mutant mice [25]. However, when colitis was induced by rectal administration of 2,4,6-trinitrobenzene sulfonic acid, pretreatment with an agonistic CD137 antibody reduced IBD disease severity [26].

As sCD137 can inhibit CD137-CD137L signaling and is abundant in human blood, analyzing its levels may help elucidate the role of sCD137 in IBD [9,27]. To date, only one study has reported serum sCD137 levels in IBD, finding levels similar in patients with Crohn’s disease and ulcerative colitis compared with controls [13]. However, serum sCD137 levels are altered in patients with other autoimmune diseases. For example, patients with type 1 diabetes had lower serum sCD137 levels than controls [17]. Notably, sCD137 effectively treated diabetes in nonobese diabetic mice [17]. Conversely, patients with rheumatoid arthritis had increased serum sCD137 levels [12]. Moreover, sCD137 levels were also higher in the serum of mice with systemic lupus [28].

Primary sclerosing cholangitis (PSC) is a chronic cholestatic liver disease characterized by inflammation and fibrotic remodeling of the bile ducts, which can progress to advanced fibrosis and cirrhosis. A substantial proportion of patients with PSC have concomitant IBD, defining the distinct PSC-IBD phenotype [29]. Apart from orthotopic liver transplantation, no curative treatment is currently available, and ursodeoxycholic acid is mainly used to improve symptoms [30]. Despite ongoing progress, the pathogenesis of PSC remains incompletely understood, and robust noninvasive biomarkers for diagnosis and disease characterization remain lacking [31,32]. Anti-neutrophil cytoplasmic antibodies may be detected in PSC, but their diagnostic value is limited by poor specificity, as they also occur in a range of other autoimmune diseases [33].

Serum sCD137 is an immunoregulatory molecule that has been reported to be elevated in liver cirrhosis related to chronic hepatitis C virus (HCV) infection or alcohol-associated liver disease [15]. However, its relevance in PSC, including more advanced disease stages, has not been defined. Moreover, serum sCD137 has not, to our knowledge, been studied in PSC-IBD. We therefore assessed serum sCD137 levels in patients with IBD, PSC-IBD, and PSC without underlying IBD to determine whether sCD137 is associated with intestinal inflammation, chronic liver injury, or both. We compared circulating sCD137 levels between control and disease groups and evaluated whether sCD137 correlated with markers of intestinal inflammation, liver dysfunction, and fibrosis. The serum of patients with MASLD was also analyzed as a disease control group.

## 2. Materials and Methods

### 2.1. Patients and Controls

Patients were recruited from both the inpatient and outpatient clinics of our university hospital between 6 December 2021 and 31 January 2025. Only those patients capable of providing informed consent were included in the study. Diagnosis of IBD and PSC was established according to clinical, laboratory, histological, radiological, and endoscopic criteria [34,35,36]. As histological and endoscopic evaluations were not performed in most cases at the time of blood collection, fecal calprotectin was used as a disease activity measure. Fecal calprotectin has demonstrated strong performance as a marker for predicting disease outcomes and tracking disease activity [37]. Fecal calprotectin remains the only biomarker shown to forecast disease recurrence in clinical studies and provides a more accurate and comprehensive assessment than endoscopically derived histological scoring systems [37].

Pregnancy, known coagulopathy, and cholangiocarcinoma were exclusion criteria. Almost all patients with PSC were treated with ursodeoxycholic acid. Patients with IBD were treated with corticosteroids (21 patients), mesalazine (20 patients), anti-interleukin 12/23 antibody therapy (19 patients), anti-TNF antibodies (24 patients), and azathioprine (8 patients).

The serum of 26 patients with ultrasound-diagnosed liver steatosis was also analyzed. The patients were 55 years old (range 38–80) and included 12 females and 14 males. Sex did not differ between the cohorts, but patients with steatosis were older than patients with IBD and patients with PSC. Alcohol and drugs causing steatosis were excluded, and patients were diagnosed with metabolic dysfunction-associated steatotic liver disease (MASLD). Whether these patients had simple steatosis or metabolic dysfunction-associated steatohepatitis was not determined, as this requires a liver biopsy.

Moreover, the serum of 78 healthy controls was collected. The controls were hospital staff, students, and the spouses of patients, students, and staff.

### 2.2. ELISA

The ELISA used to measure human sCD137 in serum was obtained from R&D Systems (Wiesbaden, Nordenstadt, Germany; Cat # DY838). The serum samples were diluted twofold and measured in duplicate; the mean values were used for calculations.

### 2.3. Liver Stiffness

Liver stiffness was evaluated in the patients utilizing acoustic radiation force impulse elastography. Each examination comprised eight measurements in different regions of the liver, approximately 5 cm below the liver capsule and 2 cm beneath Glisson’s capsule. The median of these values was calculated to minimize inter-sample variability. A standardized protocol for patient positioning and breathing, in accordance with established guidelines, was adhered to throughout the procedure [38,39]. Imaging and data acquisition were conducted using the Logiq™ E10 system (GE HealthCare, Munich, Germany). Liver stiffness measurement was performed within six months of blood sample collection. Since an increase in liver stiffness within one year is minimal in patients with PSC/PSC-IBD [40], this timeframe is considered appropriate.

### 2.4. Diagnosis of Steatosis

Hepatic steatosis was diagnosed on ultrasound when the liver parenchyma appeared more echogenic than the adjacent right renal cortex, resulting in a characteristically bright liver pattern. Additional sonographic features could include posterior beam attenuation, reduced visualization of the diaphragm, and diminished clarity of the portal vein walls. The degree of steatosis was not assessed using visual grading systems, such as the semi-quantitative method described by Ibacahe et al. [41], nor was it measured with quantitative ultrasound techniques.

### 2.5. Statistics

According to the Shapiro–Wilk test, serum sCD137 levels in patients with IBD were not normally distributed (*p* < 0.001). Therefore, data are presented as boxplots (minimum, maximum, first and third quartiles, and the median). Small circles and asterisks are used to indicate outliers. Mild outliers, which fall between 1.5 and 3 interquartile ranges from the box, are represented by circles. In contrast, extreme outliers, which lie more than 3 interquartile ranges from the box, are indicated by asterisks. The Mann–Whitney U-test, multiple linear regression, receiver operating characteristic curve, and Kruskal–Wallis test, as well as Spearman correlation (IBM SPSS Statistics 31.0, released by IBM Corp., Armonk, NY, USA, in 2025), were used for analysis, and a value of *p* < 0.05 was regarded as significant.

## 3. Results

### 3.1. Serum sCD137 Levels of Patients with IBD

Serum sCD137 was measured in 50 patients with Crohn’s disease (CD), 27 patients with ulcerative colitis (UC), and 78 controls (46 females and 32 males, aged 54 (23–80) years). Sex distribution between patients and controls was similar (*p* = 0.65).

**Figure 1:**
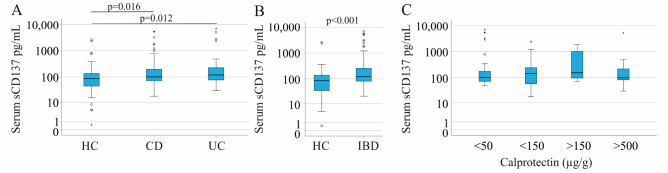
Serum sCD137 levels of patients with inflammatory bowel disease (IBD) and controls: (**A**) serum sCD137 of controls (HC), patients with Crohn’s disease (CD), or ulcerative colitis (UC); (**B**) serum sCD137 of HC and patients with IBD (CD and UC combined), ages < 60 years; (**C**) serum sCD137 levels of patients with IBD stratified for fecal calprotectin levels. Small circles and asterisks are used to indicate outliers.

Serum sCD137 levels showed substantial variation across all cohorts. Median (minimum–maximum) levels were 100.7 (17.5–5534.1) pg/mL in CD, 119.1 (29.6–7029.8) in UC, and 87.4 (0.5–2969.5) in controls (Figure 1A). Patients had higher serum sCD137 levels than controls, but levels were similar between patients with CD and those with UC (Figure 1A).

Patients with CD (*p* = 0.074) or UC (*p* = 0.279) had a similar age to controls. When combining CD and UC patients, the patients with IBD had higher sCD137 than the controls (*p* = 0.003) but were also younger than the controls (*p* = 0.012). When patients and controls aged below 60 years were compared, the ages of the 67 patients and 52 controls were similar (*p* = 0.919), and sCD137 was elevated in IBD (*p* < 0.001) (Figure 1B).

The area under the receiver operating characteristic curve (AUROC) for distinguishing patients with IBD from controls was 0.640 ± 0.044 (*p* = 0.002), indicating that serum sCD137 levels are not suitable for diagnosing IBD.

### 3.2. Serum sCD137 Levels in Relation to Disease Severity, Symptoms, and Therapy in IBD

To evaluate the association between serum sCD137 levels and disease severity, patients were stratified by fecal calprotectin levels. However, the 38 patients with low fecal calprotectin (<50 µg/g), the 18 patients with calprotectin levels > 50 µg/g and <150 µg/g, the 9 patients with calprotectin levels > 150 µg/g and <500 µg/g, and the 10 patients with high fecal calprotectin levels (>500 µg/g) had similar serum sCD137 levels (Figure 1C). Fecal calprotectin levels of two patients were not documented.

**Figure 2:**
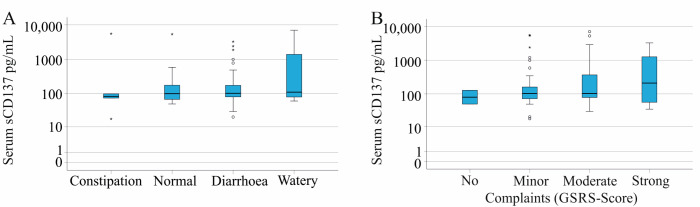
Serum sCD137 levels of patients with inflammatory bowel disease (IBD) in relation to stool consistency and disease symptoms: (**A**) serum sCD137 levels of IBD patients with constipation, normal stool, diarrhea, and watery stool; (**B**) serum sCD137 levels of patients with no, minor, moderate, or strong complaints. Small circles and asterisks are used to indicate outliers.

Serum sCD137 did not correlate with C-reactive protein or fecal calprotectin levels (*p* > 0.05 for both). There was no association between serum sCD137 and stool consistency, as assessed by patients using the Bristol Stool Chart (Figure 2A). IBD patients with constipation (5 patients), normal stool (19 patients), diarrhea (33 patients), and watery stool (8 patients) had similar serum sCD137 levels. The stool consistency of 12 patients was unknown.

The Gastrointestinal Symptom Rating Scale (GSRS), used to evaluate general well-being, abdominal pain, and daily stool frequency, showed no correlation with serum sCD137 levels. Serum sCD137 levels of patients with no (2 patients), minor (43 patients), moderate (25 patients), or strong (4 patients) complaints were similar (data of 3 patients were not recorded) (Figure 2B).

Time since diagnosis was documented for 69 IBD patients but did not correlate with sCD137 serum levels (r = −0.110, *p* = 0.368).

Twenty-one of our CD patients had a fistula, but patients with and without this severe complication had similar sCD137 in serum (*p* = 0.959).

In patients with CD, disease localization was ileocecal in 9, ileocecal with other parts of the gastrointestinal tract affected in 38, and not involving the ileocecal region in 3; however, this was not associated with sCD137 (*p* = 0.453). This was also not significant in UC (*p* = 0.412), in which 17 patients had pancolitis, 4 had left-sided colitis, 4 had proctosigmoidosis, and 1 had proctitis (disease localization for 1 patient was not documented).

A history of surgery was present in 13 patients, which was not associated with serum sCD137 levels (*p* = 0.878).

**Figure 3:**
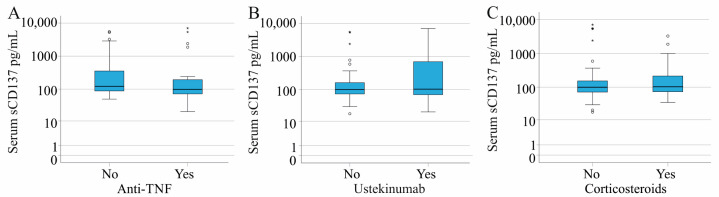
Serum sCD137 levels of patients with inflammatory bowel disease (IBD) in relation to current medication: (**A**) serum sCD137 levels of IBD patients with (Yes) and without (No) anti-TNF therapy; (**B**) serum sCD137 levels of IBD patients with (Yes) and without (No) ustekinumab; (**C**) serum sCD137 levels of IBD patients with (Yes) and without (No) corticosteroid therapy. Small circles and asterisks are used to indicate outliers.

The 19 IBD patients treated with ustekinumab (*p* = 0.682) and the 24 patients treated with anti-TNF antibodies (*p* = 0.290) had sCD137 levels similar to those of patients receiving other therapies (Figure 3A,B). This was also the case for corticosteroids (21 patients, *p* = 0.739) (Figure 3C).

Multiple linear regression analysis showed that serum sCD137 levels are not predicted by medication (F(3,58) = 1.238, *p* = 0.304 (*p* = 0.468 for TNF, *p* = 0.089 for ustekinumab, and *p* = 0.413 for corticosteroid therapy)).

### 3.3. Serum sCD137 Levels of Patients with PSC, PSC-IBD, and MASLD

This analysis included 11 patients with primary sclerosing cholangitis (PSC) without IBD and 33 patients with PSC who had associated IBD (PSC-IBD). Patients with PSC had lower C-reactive protein levels than those with IBD or PSC-IBD. Alanine aminotransferase levels in patients with PSC-IBD were higher than in patients with IBD. The levels of alkaline phosphatase, bilirubin, and aspartate aminotransferase were higher in patients with PSC or PSC-IBD than in patients with IBD (Table 1).

**Figure 4:**
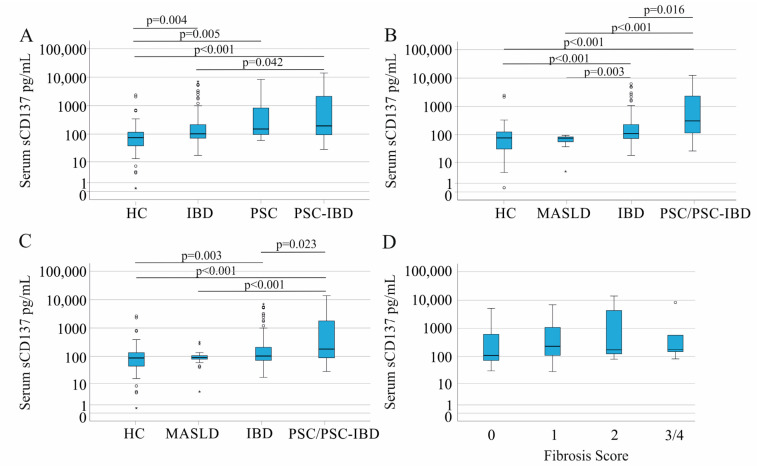
Serum sCD137 of controls, patients with inflammatory bowel disease (IBD), patients with primary sclerosing cholangitis (PSC), patients with PSC-IBD, and patients with metabolic dysfunction-associated steatotic liver disease (MASLD): (**A**) serum sCD137 of controls, patients with IBD, PSC, and PSC-IBD; (**B**) serum sCD137 levels of controls, patients with MASLD, patients with IBD, and patients with PSC/PSC-IBD, all aged below 60 years; (**C**) serum sCD137 levels of controls, patients with MASLD, patients with IBD, and patients with PSC/PSC-IBD in the entire cohort; (**D**) serum sCD137 levels of patients with PSC/PSC-IBD stratified for fibrosis score, where those with F3/F4 fibrosis were grouped together. Small circles and asterisks are used to indicate outliers.

Patients with PSC and PSC-IBD had similar serum sCD137 levels, which were higher than in the control group. Patients with PSC-IBD also had higher serum sCD137 levels than patients with IBD (Figure 4A).

The AUROC to discriminate patients with PSC-IBD from patients with IBD was 0.630 ± 0.062 (*p* = 0.035), showing that sCD137 is not appropriate for the diagnosis of PSC-IBD in patients with IBD.

Patients with PSC and PSC-IBD were grouped together for further analysis because their sCD137 levels were similar (Figure 4A). The AUROC for discriminating between PSC/PSC-IBD and controls was 0.751 ± 0.047 (*p* < 0.001). This value is acceptable for use as a complementary biomarker in the diagnosis of PSC/PSC-IBD.

Patients with PSC/PSC-IBD were similar in age to those with IBD (*p* = 0.949), but younger than the control group (*p* = 0.028). Among patients younger than 60 years, there was no difference in age between the cohorts (*p* = 0.491), and sCD137 was higher in PSC/PSC-IBD patients (35 patients) than in controls (52; *p* < 0.001) and IBD patients (67; *p* = 0.012).

Because they had similar sCD137 levels, patients with PSC and PSC-IBD were also grouped together for correlation analysis. In this cohort, sCD137 did not correlate with C-reactive protein, aminotransferase levels, gamma-glutamyltransferase, alkaline phosphatase, the MELD score, or bilirubin (all *p* > 0.05).

Serum sCD137 levels were also measured in 26 patients with MASLD to assess whether elevated levels are associated with liver disease in general. These patients had lower alkaline phosphatase levels than patients with PSC or PSC-IBD but otherwise had similar levels of liver disease severity markers (Table 1). Patients with MASLD had the highest body mass index (Table 1).

Because patients with MASLD were older than those in the other groups (Table 1), only individuals younger than 60 years were included in this analysis. In this age-restricted subgroup, age did not differ significantly among the groups: 67 patients with IBD, 35 with PSC/PSC-IBD, 52 healthy controls, and 16 with MASLD (*p* = 0.054). Serum sCD137 levels in patients with MASLD were comparable to those in healthy controls but lower than in patients with IBD and PSC/PSC-IBD (Figure 4B). These findings were consistent with the differences observed in the overall cohort (Figure 4C). In the MASLD cohort, serum sCD137 did not correlate with laboratory values of liver disease (all *p* > 0.05).

Fibrosis grade in patients with PSC/PSC-IBD was documented in 41 patients (11 scored 0, 17 scored 1, 8 scored 2, 1 scored 3, and 4 scored 4), but this was not associated with serum sCD137 levels (Figure 4D).

### 3.4. Confounding Variables

In patients with IBD and PSC/PSC-IBD, sCD137 did not correlate with BMI and was negatively correlated with age (r = −0.335, *p* = 0.003; r = −0.592, *p* < 0.001, respectively). No correlation between serum sCD137 levels and age was observed in the controls (*p* > 0.05).

Sex differences were not observed in controls (*p* = 0.139), patients with IBD (*p* = 0.251), or patients with PSC/PSC-IBD (*p* = 0.095).

Multiple linear regression in patients with IBD showed that age, BMI, and sex did not predict serum sCD137 levels (F(3,61) = 0.822, *p* = 0.487). Multiple linear regression in patients with PSC-IBD/PSC showed that age, BMI, and sex did not predict serum sCD137 levels (F(3,24) = 1.566, *p* = 0.223).

## 4. Discussion

This study shows that serum sCD137 levels are increased in patients with IBD and are further elevated in patients with PSC/PSC-IBD. Notably, serum sCD137 was not associated with markers of systemic or intestinal inflammation in IBD and did not increase with fibrosis stage in PSC/PSC-IBD. Together, these findings support sCD137 as a disease-associated systemic immune marker, with the strongest signal in PSC-associated disease.

In patients with HCV infection, serum sCD137 has been reported to correlate positively with non-invasive fibrosis scores, including the Fibrosis-4 score, and patients with ultrasound-diagnosed cirrhosis had higher serum sCD137 levels than those without cirrhosis [15]. In patients with sepsis, who also had higher plasma sCD137 levels than controls, plasma sCD137 tended to be higher in those with liver cirrhosis, although this trend did not reach significance [27]. In contrast, in our PSC/PSC-IBD cohort, serum sCD137 was not associated with higher fibrosis scores and did not correlate with routine measures of liver disease. This indicates that elevated serum sCD137 in PSC/PSC-IBD is unlikely to reflect liver disease activity. Patients with MASLD had normal sCD137 levels, which were lower than in patients with PSC/PSC-IBD, suggesting that liver injury per se is not the main underlying factor for higher systemic sCD137 levels. Similarly, in IBD, serum sCD137 was not associated with C-reactive protein or fecal calprotectin and did not increase in patients with more active disease. Clinical symptoms, including diarrhea and abdominal pain, were likewise not linked to higher sCD137 levels. Moreover, serum sCD137 was not associated with disease duration, disease localization, or fistula, a serious complication of these patients. This argues against an association with immune activation and immunoregulation, both of which change with disease activity [42]. The observation that serum sCD137 remains relatively stable across different levels of disease activity suggests that it may reflect a more persistent immunological state rather than a fluctuating inflammatory burden.

Luu et al. proposed that sCD137 may exacerbate Th17-driven autoimmune diseases [9], and the elevated serum sCD137 levels observed in our cohort are consistent with this concept. Agonistic CD137 antibodies have been shown to improve disease severity in IBD [26], suggesting that higher levels of sCD137, which can block CD137-CD137L signaling [9], may contribute to disease persistence. CD137 stimulation enhances T cell function, which may be reduced by higher sCD137 levels, eventually contributing to T cell exhaustion, a common phenomenon in chronic inflammatory and autoimmune diseases [43,44].

The increased serum sCD137 levels observed in IBD and PSC support the concept that CD137 is induced in these conditions. CD137 expression in healthy tissues is generally induced only after cellular activation, and this activation-dependent pattern has been observed across all normal tissues examined [9]. However, this observational study cannot identify the tissue source of the higher sCD137 levels in IBD and PSC. Comparison of cirrhotic and non-fibrotic liver tissue has shown similar CD137 protein expression, suggesting that hepatic CD137 expression is not closely related to fibrosis stage [15]. Whether this also applies in PSC/PSC-IBD remains to be determined.

Almost all of our patients with PSC/PSC-IBD were given ursodeoxycholic acid. A recent study has reported that serum sCD137 levels of controls and patients with primary biliary cholangitis were similar [45]. In that cohort, patients who responded to ursodeoxycholic acid and those who did not respond had similar plasma sCD137 levels [45]. This argues against the effect of ursodeoxycholic acid therapy on circulating sCD137 levels. Patients with IBD receiving biologics and/or corticosteroids had serum sCD137 levels comparable to those of patients treated with other medications, and we found no significant difference between the groups. However, the study may have been underpowered to detect such differences, particularly given the substantial interindividual variability in serum sCD137 levels.

To date, only Seidel has examined serum sCD137 levels in IBD, in a small cohort comprising five patients with UC, five with CD, and 13 controls [13]. In that study, serum sCD137 levels in patients were reported to be within the normal range. As in our study, Seidel also observed a wide range of sCD137 concentrations in healthy controls, from 0 to 2200 pg/mL [13]. The relatively small cohort may have limited the ability to detect significant differences [13]. Importantly, Seidel found no difference in sCD137 levels between CD and UC [13], a finding that is confirmed in our substantially larger cohort.

Experimental studies have shown that CD137-expressing T cells do not contribute to IBD [24], whereas treatment with an agonistic CD137 antibody before disease initiation reduces IBD severity [26]. A similar effect has also been reported in other autoimmune diseases [46]. Therefore, we hypothesize that higher sCD137 levels, which inhibit CD137 activity [7], might contribute to IBD development but not to progression or severity. Individuals at risk for IBD could be screened for sCD137 levels to determine whether disease onset and elevated serum sCD137 levels occur simultaneously. Based on current data, agonistic CD137 antibody therapy may not be effective for inducing remission in IBD or for preventing fibrosis progression in PSC/PSC-IBD, as it is unlikely to be associated with disease progression. This study argues against the diagnostic and stratification value of sCD137 in IBD. Whether it is an additional marker for PSC diagnosis has to be evaluated in treatment-naïve patients with early PSC.

The interpretation of circulating protein biomarkers can be confounded by sex, age, and BMI. In the present study, however, serum sCD137 showed no sex-specific differences in controls, patients with IBD, or patients with PSC/PSC-IBD. Similarly, sex differences in circulating sCD137 were not observed in controls, sepsis patients [27], or HCV patients [15]. In HCV, sCD137 did not correlate with BMI, in line with our findings in IBD and PSC/PSC-IBD. Regarding age, both positive and null associations with circulating sCD137 have been reported [15,27]. In our study, sCD137 was negatively associated with age in the patient cohorts but not in controls, indicating that the relationship between age and circulating sCD137 warrants further investigation. However, in age-matched subcohorts, serum sCD137 levels remained higher in IBD and PSC/PSC-IBD than in controls and/or patients with MASLD.

This study has limitations. The small number of patients with PSC without underlying IBD prevented the identification of potential differences with IBD and/or PSC-IBD. It should be noted that PSC-IBD and PSC are often combined in a single study cohort [47,48], as in the current study. The study may also have been underpowered to detect medication effects, particularly given the substantial interindividual variability in serum sCD137 levels. Blood samples were collected at a single time point, and sCD137 was not assessed longitudinally before or during therapy. In IBD, sCD137 was not associated with disease severity assessed by fecal calprotectin and serum C-reactive protein levels. Thus, we do not expect sCD137 levels to change with improvement in inflammation, a suggestion to be confirmed in longitudinal studies. However, histological and endoscopic evaluations were not performed in most cases at the time of blood collection, which may weaken our conclusion that serum sCD137 is not related to disease severity. Because this was an observational study, the tissue source and the pathophysiological role of elevated serum sCD137 in IBD and PSC-IBD could not be addressed. However, the clear increase in circulating sCD137 across these disease groups, particularly in PSC-associated disease, provides a strong rationale for future mechanistic and prospective studies.

## 5. Conclusions

Serum sCD137 is elevated in IBD and further increased in PSC-associated disease. While sCD137 did not track with inflammatory activity or fibrosis stage, its stable increase in IBD and PSC/PSC-IBD supports its relevance to disease-associated immune dysregulation.

## Figures and Tables

**Table 1:** Characteristics of IBD patients, PSC-IBD, PSC patients, and MASLD patients for analysis of serum sCD137. Data are presented as medians, minimums, and maximums. Statistical test used: Kruskal–Wallis test (alanine aminotransferase (ALT), alkaline phosphatase (AP), aspartate aminotransferase (AST), gamma glutamyltransferase (gamma GT), model for end-stage liver disease (MELD)); *, ^%^, ^b^, ^c^ *p* < 0.05, **, ^&&^, ^aa^, ^cc^ *p* < 0.01; ***, ^&&&^, ^aaa^, ^bbb^ *p* < 0.001.

Characteristics	IBD	PSC-IBD	PSC	MASLD
Number (females/males)	77 (37/40)	33 (13/20)	11 (5/6)	26 (12/14)
Age (years)	45 (20–70) ^bbb^	43 (18–67) *^,aaa^	54 (26–70) *	55 (38–80) ^aaa,bbb^
Body mass index (kg/m^2^)	24.6 (15.5–44.3) ^b^	23.9 (17.0–41.8) ^aa^	19.9 (18.0–21.8) ^c^	26.7 (22.1–43.40) ^aa,b,c^
C-reactive protein (mg/L)	2 (0–144) *	3 (0–93) ^%^	0 (0–0) *^,%^	not determined
Fecal calprotectin (µg/g)	54 (0–3889)	48 (0–999) ^13^	not determined	not determined
AST (U/L)	24 (12–41) *^,&&&,bbb^	41 (16–161) ^&&&^	31 (15–177) *	36 (28–96) ^bbb^
ALT (U/L)	20 (7–62) ^&&&,b^	37 (7–205) ^&&&^	27 (5–89)	24 (12–52) ^b^
Gamma GT (U/L)	25 (10–100) **^,&&,b^	50 (10–1700) ^&&^	78 (11–345) **	48 (8–752) ^b^
AP (U/L)	64 (40–142) ***^,&&&^	127 (35–447) ^&&&,aaa^	158 (70–537^)^ ***^,cc^	64 (48–516) ^aaa,cc^
Bilirubin (mg/dL)	0.50 (0.10–1.90) **^,&&^	0.65 (0.20–21.30) ^&&^	0.80 (0.40–14.00) **	0.60 (0.20–21.30)
MELD Score	not determined	6 (6–15)	7 (6–20)	not determined
Diagnosis (years)	14 (0–42) 69 patients	11 (0–48) 15 patients	not determined	not determined
Type 2 diabetes	1	1	0	2
Hypertension	8	2	0	2

## Data Availability

The data are included in the manuscript. Original data can be obtained from the corresponding author upon request.

## Abbreviations

The following abbreviations are used in this manuscript:

ALT	Alanine aminotransferase
AP	Alkaline phosphatase
AST	Aspartate aminotransferase
AUROC	Area under the receiver operating characteristic curve
BMI	Body mass index
CD	Crohn’s disease
DSS	Dextran sodium sulfate
Gamma GT	Gamma-glutamyltransferase
HCV	Hepatitis C virus
IBD	Inflammatory bowel disease
IL	Interleukin
MELD	Model for end-stage liver disease
sCD137	Soluble CD137
UC	Ulcerative colitis
PSC	Primary sclerosing cholangitis
TNF	Tumor necrosis factor
